# Artificial Pancreas or Novel Beta-Cell Replacement Therapies: a Race for Optimal Glycemic Control?

**DOI:** 10.1007/s11892-018-1073-6

**Published:** 2018-09-24

**Authors:** Michiel F. Nijhoff, Eelco J. P. de Koning

**Affiliations:** Department of Medicine, Division of Nephrology and Transplantation, Division of Endocrinology and Metabolism, Leiden University Medical Centre, PO Box 9600, 2300 RC Leiden, the Netherlands

**Keywords:** Diabetes mellitus, Stem cells, Bionic pancreas

## Abstract

**Purpose of Review:**

New treatment strategies are needed for patients with type 1 diabetes (T1D). Closed loop insulin delivery and beta-cell replacement therapy are promising new strategies. This review aims to give an insight in the most relevant literature on this topic and to compare the two radically different treatment modalities.

**Recent Findings:**

Multiple clinical studies have been performed with closed loop insulin delivery devices and have shown an improvement in overall glycemic control and time spent in hypoglycemia. Beta-cell transplantation has been shown to normalize or greatly improve glycemic control in T1D, but the donor organ shortage and the necessity to use immunosuppressive agents are major drawbacks. Donor organ shortage may be solved by the utilization of stem cell-derived beta cells, which has shown great promise in animal models and are now tested in clinical studies. Immunosuppression may be avoided by encapsulation.

**Summary:**

Closed loop insulin delivery devices are promising treatment strategies and are likely to be used in clinical practice in the short term. But this approach will always suffer from delays in glucose measurement and insulin action preventing it from normalizing glycemic control. In the long term, stem cell-derived beta cell transplantation may be able to achieve this, but wide implementation in clinical practice is still far away.

## Introduction

Over 400 million people worldwide suffer from diabetes mellitus and more than half a million are children below the age of 15 years. The majority of children suffer from type 1 diabetes (T1D), while in adults, the prevalence is up to 10% of the total population (depending on age and geographic distribution) [1, 2]. The current treatment for T1D is exogenous insulin, either through multiple daily injections or continuous subcutaneous insulin infusion (CSII). Neither therapy can prevent marked glycemic variability that is associated with an increased risk of hypoglycemia, necessitating higher glucose targets to prevent severe hypoglycemic events. However, higher average glucose concentrations are directly linked to diabetes-related complications and cardiovascular disease [3•, 4•, 5].

Several treatment options to improve glycemic variability and control have been explored in the past decades. The advancement of continuous glucose sensors and new insulin pump devices has sparked the development of currently used hybrid closed loop devices. These devices utilize continuous glucose measurement (CGM) to regulate insulin administration through a control algorithm, but still require preprandial active insulin dosing. Closed loop devices that do not require active adjustment of insulin dosing are considered artificial pancreases, and several of these systems are currently being tested [6].

A cell-based approach to achieve optimal glycemic control is beta-cell transplantation, either through whole organ or islet transplantation. It normalizes or at least greatly improves glycemic control, often without the need for exogenous insulin [7]. Novel cell replacement therapies using human pluripotent stem cell (hPSC)-derived insulin-producing cells have recently been tested in early clinical studies [8••].

In this review, we will assess the potential and current status of the artificial pancreas and novel beta cell replacement strategies. Is there a race for optimal glycemic control between these two different treatment strategies?

## Artificial Pancreas

Technology is playing an increasingly important role in the management of T1D. Not until the 1960s, capillary blood glucose meters were introduced, providing progressively more accurate point-of-care glucose measurements [9]. In the late 1990s, CGM devices entered clinical practice, allowing for real-time interstitial glucose measurements, thereby improving glycemic stability [10]. Continuous subcutaneous insulin infusion (CSII) using small pumps had already been introduced in the 1970s [11, 12]. The CSII devices are continuously being improved with regard to size and function. Current devices offer several insulin administration options, such as variable basal rates and bolus infusions. There are pumps with separate infusion sets and also patch pumps that can be placed directly onto the skin [13]. Communication between the CGM and CSII devices was the logical next step.

### The Artificial Pancreas

Systems that automatically turn off insulin administration based upon continuous interstitial glucose measurements (low glucose suspend and predictive low glucose suspend systems) were the first to be used in clinical practice. While automatic stopping and resuming insulin administration has been shown to be safe in patients with T1D [13], it took some time for systems that automatically administer a bolus dose of insulin. Recently, a hybrid closed loop system has entered the market (Minimed™ 670G, Medtronic©). With this system, continuous, sensor-augmented, and automated subcutaneous insulin delivery is combined with user actions around meal times (carbohydrate announcement, acceptance of bolus recommendation) to mitigate postprandial glycemic variation [9].

The next step was a true closed loop system that also accounts for post-prandial glucose excursions and variability in glucose concentrations caused by other factors such as exercise and hormonal changes. This system could be considered an artificial pancreas. Recent studies demonstrate the feasibility and efficacy of a closed loop system in a free-living, unsupervised setting (Table 1). The earliest studies focused on automated nighttime control only. In these studies, a beneficial effect was demonstrated on time spent in a hypoglycemic range and time spent in a normoglycemic range during the treatment period [14, 15•]. Tabit et al. showed the feasibility of an artificial pancreas in 33 patients with T1D that were not supervised [16••]. A modest reduction in glycated hemoglobin (HbA1c) was achieved (4 mmol/mol Hb (0.3%)) after 12 weeks. The time spent in hypoglycemia was reduced by almost 40%, and the only severe hypoglycemic event occurred during the intervention phase (due to connectivity problems of the device). Garg et al. tested the same system in 124 adult and adolescent patients with T1D [17••]. Patients were treated with the bihormonal closed loop system for 3 months after a 2-week run-in period. Their results were similar to the previous study, and adverse events were not reported.

**Table 1:** Landmark studies on the artificial pancreas in a continuous, free-living setting

Author	Journal, year	Study design	Intervention	Patients^a^	Outcome^b^	Limitations
Russell et al.	N Engl J Med 2014	Random-order crossover trial	5 day bihormonal artificial pancreas versus CSII	52 patients, 20 adults and 32 adolescents, with an HbA1c of 38 to 103 mmol/mol Hb (5.6–11.6%)	− 44% time spent in hypoglycaemia (< 3.9 mmol/L) + 12% time spent in normoglycemia Average glucose fell from 8.9 mmol/L to 7.4 mmol/L	Very short treatment duration Exclusion of patients with reduced hypoglycemia awareness, insulin resistance
Thabit et al.	N Engl J Med 2015	Randomized crossover trial	12 week continuous use of closed-loop insulin delivery system versus sensor-augmented pump	33 patients, all adult with an HbA1c of 58–86 mmol/mol Hb (7.5–10%)	HbA1c − 4 mmol/mol Hb − 39% time spent in hypoglycaemia (< 3.5 mmol/L) + 16% time in normoglycemia 1 severe hypoglycemic event during intervention phase	Short follow-up Exclusion of patients with reduced hypoglycemia awareness, a previous severe hypoglycemic event, insulin resistance
El-Khatib et al.	Lancet 2017	Randomized crossover trial	11 day bihormonal artificial pancreas versus usual care	43 patients, all adult	− 68% time spent in hypoglycaemia (< 3.3 mmol/L) + 17% time spent in normoglycemia Average glucose fell from 9.0 mmol/L to 7.8 mmol/L Nausea score increased from 0.05 to 0.52 on visual scale	Short treatment duration
Garg et al.	Diabetes Technol Ther 2017	Single-arm prospective trial	3 months continuous use of closed-loop insulin delivery system	124 patients, 94 adults and 30 adolescents, with an HbA1c < 86 mmol/mol Hb (10%)	HbA1c − 5 mmol/mol Hb − 47% time spent in hypoglycemia (< 3.9 mmol/L) + 12% time in normoglycemia	No control treatment Short follow-up Exclusion of patients with severe hypoglycemic events, poorly controlled diabetes No report on adverse events

^a^All patients were diagnosed with type 1 diabetes mellitus at least a year before and were on long term (> 6 months) subcutaneous insulin pump therapy

^b^Data reported here is for the adult population. Normoglycemia: glucose 3.9–10 mmol/L

When subcutaneous glucagon infusion is added to the closed loop system with its own control algorithm, a bi-hormonal closed loop system is created. Russell et al. treated 52 patients with T1D with 5 days of insulin pump and 5 days of bi-hormonal artificial pancreas treatment [18••]. In these patients, time spent in hypoglycemia was reduced by over 40%, and time spent in normoglycemia was increased by 13%. Mean glucose fell from 8.9 to 7.4 mmol/L. No severe hypoglycemic events occurred. El-Khatib et al. [19••] further investigated this bi-hormonal device. They included 43 patients with T1D comparing the bi-hormonal device with usual care (conventional or sensor-augmented pump therapy). The intervention phase lasted 11 days and yielded similar results. These studies have confirmed that with both the insulin-only and the bi-hormonal closed loop devices, a significant reduction of time spent in hypoglycemia could be achieved without an increase in HbA1c. Importantly, all studies demonstrated a greater beneficial effect of the intervention during nighttime than during the day [16••, 17••, 18••, 19•].

### Limitations of the Artificial Pancreas

Several important limitations apply to these studies. First, patients who had a history of severe hypoglycemic events were excluded in the studies with the insulin-only closed loop systems [16••, 17••]. In some studies, patients with reduced hypoglycemia awareness and insulin resistance were also excluded [17••, 18••]. Although patients with a history of severe hypoglycemic events were not explicitly excluded in the bi-hormonal closed loop device studies, participation of these patients was not reported.

Treatment and follow-up time were generally short, especially in the bi-hormonal closed loop device studies (5 to 11 days). This makes it difficult to assess potential severe adverse effects during longer use, such as severe hypoglycemia or ketosis in case of device malfunction or loss of connectivity. Indeed, loss of connectivity has been shown to occur up to 4% of the time [19•] and can lead to severe hypoglycemic events [16••].

**Fig. 1:**
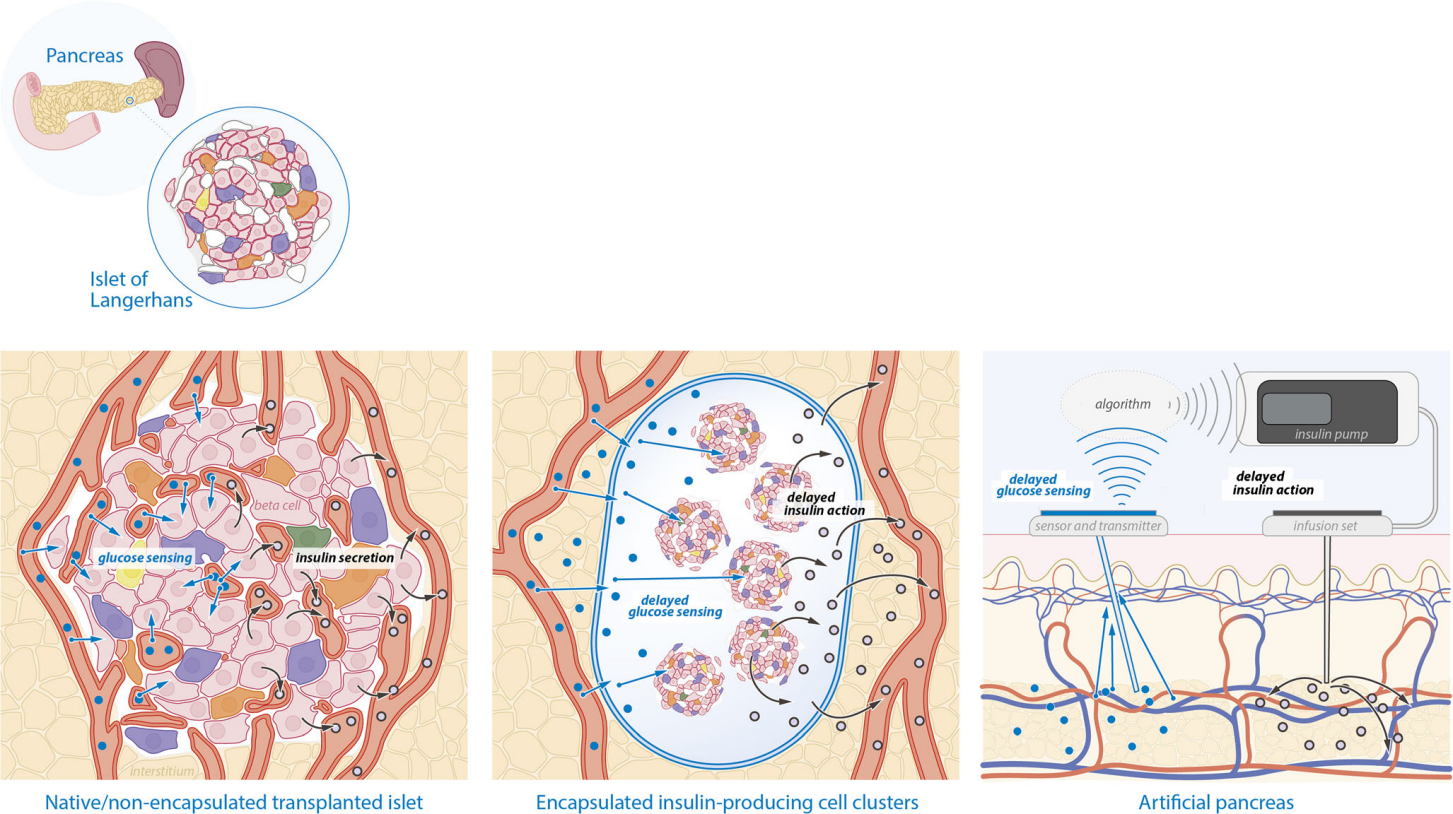
Challenges in obtaining optimal “real-time” glycemic control in artificial pancreas and encapsulated beta-cell replacement strategies. Left panel: in native pancreatic islets or transplanted islets in the liver that have been vascularized, the insulin-producing beta cells are in close proximity to the islet capillary network. Nutrients, in particular carbohydrates (blue dots), are rapidly sensed by the insulin-producing cells. Based on nutrient levels, the cells are able to immediately secrete the appropriate amount of insulin (black dots) into the islet capillaries. Middle panel: cell clusters containing insulin-producing cells (islets, beta-like cells derived from pluripotent stem cells) that are loaded into (macro)encapsulation devices before implantation in a recipient. There is no direct contact between the insulin-producing cells and capillaries. This “dead space” and limitations in transport of molecules across the macrocapsule membrane cause delayed (blood) glucose sensing and delayed insulin action. Right panel: in current artificial pancreases, there is a glucose sensor in the skin which is coupled to a transmitter that sends information about glucose concentrations to a receiver. This receiver feeds the information in a control algorithm that controls insulin delivery through an infusion set. There is a variable delay in blood glucose reporting, using interstitial glucose monitoring by a subcutaneous glucose sensor. This can also be termed “delayed (blood) glucose sensing.” There is also a delay between subcutaneous insulin administration and resorption of insulin into the blood stream causing delayed insulin action

Another important current limitation of closed loop devices is lag time. CGM systems that are currently used in clinical practice measure the glucose concentration in the interstitial fluid using enzymatic oxidation of glucose [20]. There is a variable latency that can go up to 15 min between blood glucose and interstitial fluid glucose in patients with T1D [21•] (Fig. 1). The CGM itself may have a latency period, caused by the fact that most CGMs provide a glucose measurement every 5 min that is an average of the glucose in those previous 5 min [22]. Altogether, the glucose concentration reported by CGM may reflect a variable delay of up to 20 min. There is also a variable measurement error and reduced measurement accuracy during the first few days of using the CGM. Accuracy is influenced by factors such as skin temperature, pressure, and movement [23, 24]. Although the mean average relative difference of current CGMs continues to improve and approaches only 10%, this error margin increases during rapid changes in glucose concentrations and in the hypoglycemic range [14].

It should also be noted that subcutaneously administered insulin, either by a CSII device (as part of a closed loop system) or injection, will always have a lag time until its pharmacological action (Fig. 1). Even the fastest acting subcutaneous insulin (faster-acting insulin aspart) takes up to 45 min to achieve the maximum concentration after a bolus and increases serum insulin levels up to 3 h after injection [25•]. This latency can vary depending on many factors, such as skin temperature, physical activity (which also affects insulin sensitivity) [26], location of inserted cannula, and local fibrous tissue formation [14]. Modern control algorithms try to account for the delay of insulin action and glucose reporting in the insulin delivery model [9, 15]. Despite improvements, the variable delay as mentioned above still hampers complete glycemic control, as is evidenced by the persistence of daytime glycemic variability [15].

### Practical Issues

Some important practical issues regarding the artificial pancreas are also not yet solved. For example, multiple calibrations per day are still required for some of the more common CGM devices, necessitating daily finger pricks. Recent developments in flash glucose monitoring devices have shown that this problem may be solved [27•]. Also, meals remain difficult to adjust for. Many of the closed-loop devices require meal announcements but still struggle to improve postprandial glucose control [16••, 17••, 18••, 19•, 28]. Besides the CGM and CSII lag time, this is probably an important reason that 17–29% of the time is still spent in a hyperglycemic range (> 10 mmol/L).

A further practical issue is that of acetaminophen. This common pain killer can lead to falsely elevated glucose concentrations (up to 3.3 mmol/L) due to increased oxidation at the sensing electrode [29]. This may be especially dangerous in the low-normal to hypoglycemic range. However, excluding patients from using acetaminophen will limit options for pain management.

Subcutaneous glucagon infusion provides specific issues for the artificial pancreas. Importantly, glucagon has a relatively short conservation time; in the trials with a bihormonal system, users had to replace the glucagon ampule at least every day [18••, 19•]. This problem may be solved by the recent development of glucagon formulations with a longer conservation time [30–32]. In addition, a side effect of glucagon is nausea, as was shown in the study by El-Khatib, in which nausea increased tenfold in the intervention phase [19•].

## Beta-Cell Transplantation

T1D is characterized by a rapid loss of endogenous beta-cell mass, leading to insulin dependence. Restoration of endogenous insulin secretion is therefore a logical approach in the ultimate goal to achieve normal glycemic control in T1D. It is known that persistence of even a low degree of endogenous insulin secretion in patients with long-standing T1D leads to fewer (severe) hypoglycemic episodes, fewer diabetes-related complications, and a lower HbA1c [33].

Serious efforts to restore beta-cell mass have been ongoing since the early 1960s, with reports of the first successful pancreas transplantation in 1967 [34] and the first human islet transplantation performed in 1977 [35]. Pancreas transplantation proved effective in reversing T1D but requires major surgery and is associated with a high risk of adverse events, such as infection, bleeding, and thrombosis [36]. These factors limit the feasibility of whole pancreas transplantation as a standard treatment option for patients with T1D. Islet transplantation focuses on replacing only the endocrine cells of the pancreas and is associated with fewer complications, but also less favorable long-term outcome with regard to glycemic control [37]. Still, major advances in the islet transplantation field have been made, such as improved islet isolation techniques [38] and improved immunosuppressive regimens [39]. These advances have led to a higher percentage of islet transplant recipients with insulin independence [37].

Currently, research in beta-cell replacement strategies focuses on improvement of immunosuppressive regimens, alternative cell sources for beta cells, alternative transplantation sites including immunoprotective scaffolds, and improvement of long-term islet survival [40].

### Clinical Islet Transplantation

The landmark trial for clinical islet transplantation was published by Shapiro et al. in 2000 [39]. In this trial, seven participants with T1D and impaired hypoglycemia awareness and/or glycemic lability were included. All participants achieved insulin independence, with normalized HbA1c and abrogation of hypoglycemic events, thereby demonstrating the feasibility and efficacy of restoring endogenous beta-cell mass. However, long-term follow-up of a larger number of patients in this center showed that only 28% remained insulin-independent, although persistent graft function still protected against hypoglycemia and improved glycemic control [41••]. More recently, a phase 3 multi-center trial of islet transplantation in 48 participants with T1D complicated by severe hypoglycemia showed that half of the participants attained insulin independence at 1 year, while almost 90% of participants had abrogation of severe hypoglycemic events with excellent glycemic control (i.e., an HbA1c < 53 mmol/mol Hb (< 7%)) [42••]. These data indicate that while insulin independence can be achieved, most patients need to resume low-dose insulin in the presence of long-term partial graft function.

Some degree of endogenous insulin production in islet transplant recipients without insulin independence still has considerable advantageous effects on improvement of impaired hypoglycemia awareness and reduction of recurrent (severe) hypoglycemic episodes, which are important treatment goals. C-peptide concentrations as low as 200 pmol/L after islet transplantation improved glycemic variability and abrogated severe hypoglycemia [43•].

### Limitations of Islet Transplantation

The clinical studies in islet transplantation show the feasibility and efficacy of restoring endogenous insulin production through a procedure with relatively few complications, although long-term outcomes still require improvement. Two other major problems are readily apparent. First of all, potent immunosuppression is required. Immunosuppressive agents have many side effects, including infection, malignancy, deterioration of kidney function, and inhibition of islet function [44]. Furthermore, current allogeneic islet transplantation requires donor pancreata. This is a scarce source of tissue. Organ donation rates (between 1.0 and 34.8 per million people per year) are much lower than the incidence of T1D (200 per million people in Europe) [45, 46]. And as the pancreas utilization rate is also low compared to most other organs, only a small fraction of patients could be treated even if less riskful immunoevasive strategies would be developed. A potential alternative source of islets is those from animal species such as pigs. Xenotransplantation using porcine islets still has important ethical and scientific challenges, especially related to immune rejection and porcine endogenous retroviruses [47].

### Human Pluripotent Stem-Cell-Derived Beta-Like Cells

Therefore, the search for alternative scalable sources for beta cells is ongoing. Human pluripotent stem cells (hPSC), such as embryonic stem cells (ESC) and induced pluripotent stem cells (iPSC), draw most interest. Several differentiation protocols have been published that allow the differentiation of pluripotent stem cells via different stages to beta-like cells that have the capacity to cure diabetes in immunodeficient mice [48–50]. Viacyte, Inc. has been leading the way using protocols to differentiate embryonic stem cells up to a pancreatic progenitor stage before transplantation. After transplantation further maturation occurs and functional glucose-lowering effects of the cell transplant occur several weeks after transplantation in mice. Interestingly, the first-in-human trials started in 2014, in which these partially differentiated cells were encapsulated in immunoprotective macroencapsulation devices before implantation under the skin in patients with T1D [51, 52•]. Results of these trials will provide relevant information for further clinical development.

Since 2007, the induction of pluripotent stem cells from patients’ own tissue has been possible, obviating the need for human embryos [53]. This has sparked an increased interest in research into iPSC-derived beta-like cells, culminating in publications with improved protocols for differentiation of iPSC into beta-like in vitro [8••, 48••]. It was also demonstrated that skin fibroblasts taken from patients with T1D could be used in such protocols [8••]. These iPSC-derived beta-like cells have yet to be tested in human trials.

### Limitations of hPSC-Derived Beta-Like Cells

Several limitations exist with hPSC-derived beta-like cells. First, although differentiation protocols lead to hPSC-derived beta-like cells that have the capacity to cure diabetes in immunodeficient mice, these cells do not have the same “mature” insulin secretory capacity as primary human beta cells. Hence, the use of the term “beta-like cells” to describe the hPSC-derived insulin-producing cells with an “immature” phenotype. In addition, a major concern is that of tumor formation. Cell transplants derived from hPSCs have been shown to produce several types of tumors, mostly teratomas [49, 50]. In addition, while beta-like cells derived from hESC are prone to allo-rejection as these cells are “non-self”, hiPSC may be prone to auto-rejection if there still is active autoimmunity against one’s own beta cells.

These limitations have led to the development of immunoprotective macro-encapsulation devices to contain the cells within the device and to protect them from the immune system after transplantation [54]. Major issues regarding mass transport of nutrients and oxygen, insulin secretory dynamics and foreign body reactions to biomaterials remain to be solved (Fig. 1). One option is to use an “open” device that allows ingrowth of vessels into the cells in the device. While vascularization will improve oxygen availability and insulin secretory dynamics, immunosuppression is necessary and there is the potential drawback of hPSC-derived cells leaving the device. This strategy using an “open” device is currently being tested in patients with T1D with labile glycemic control [51].

Several other immunoprotective encapsulation strategies have been developed [55]. The BETA-O2 macroencapsulation device is immunoprotective and contains a special oxygen chamber. Liquid oxygen is administered daily through a subcutaneous port which allows for oxygenation of transplanted cells. In a proof-of-concept case study, it was shown that human islets within the device, which was implanted at a preperitoneal site, remained viable for approximately 10 months and retained some insulin secretory capacity without the use of immunosuppressive agents [56••]. In a subsequent study using the BETA-O2 device that was implanted in a subcutaneous site in four patients with T1D, human islets survived but little insulin secretory capacity was present and there was a profound foreign body reaction [57]. All together, these data indicate that in the fields of hPSC-derived beta-like cell and immunoprotective encapsulation strategies, many exploratory clinical studies still need to be done.

## Medical Therapy Versus Beta Cell Replacement Strategies

Both beta-cell replacement strategies and a combination of glucose sensor and insulin pump technologies offer important advances in the treatment of T1D. But there is a paucity of comparative studies between optimal medical therapy and beta-cell replacement strategies. Thompson et al. [58••] compared islet transplantation with intensive medical therapy in patients with complicated T1D. In this prospective cohort study, the patients on the waiting list for islet transplantation functioned as the control group. These patients received intensive medical care to manage their diabetes. HbA1c was 51 mmol/mol Hb (6.7%) in the transplanted patients versus 62 mmol/mol Hb (7.8%) in the control patients. It was demonstrated that the patients in the control group had a greater decline in kidney function, and more progressive retinopathy. Although this study shows that beta-cell transplantation allows for better glycemic control than optimal medical therapy, closed-loop devices were not used at the time of the study. No other direct comparative studies exist, not even between different strategies within the allogeneic transplantation field (i.e., pancreas versus islet transplantation). It should also be noted that the patients included in beta-cell replacement studies and in studies using novel glucose sensor and pump strategies have very different characteristics. In islet transplantation studies, mostly patients with impaired hypoglycemia awareness and recurrent (severe) hypoglycemic episodes are included, while these patients are often excluded in studies testing novel glucose sensors and insulin pumps.

## Conclusion

T1D is currently treated with exogenous insulin guided by glucose measurements. This strategy is lifesaving but does not prevent hypoglycemia, long-term complications, and premature mortality [3, 4]. Currently, beta-cell replacement therapies and sensor-augmented insulin pump therapy are already used in clinical practice. Each strategy has its advantages and disadvantages. And while to some it may appear there is a race for optimal glycemic control between these two strategies, thus far, there are two races, each on a different track. Studies with beta cell transplantation have focused on patients with complicated T1D and severe hypoglycemic events, while studies with sensor-augmented pump therapy have largely focused on younger patients with fewer complications.

In the second stage of the race for optimal glycemic control, novel treatment strategies will be explored, such as the artificial pancreas and hPSC-derived insulin-producing cells. It is very likely that the artificial pancreas will reach a larger patient population sooner than hPSC-derived cell transplantation. A major obstacle for achieving perfect glycemic control using the artificial pancreas is the delay in action of subcutaneously administered insulin and the lag time between blood glucose concentration and display to the patient using CGM devices that measure glucose in the interstitial fluid (Fig. 1). This will prevent the artificial pancreas from completely regulating glucose levels during unexpected glucose variation, for example after a meal. Real-time glycemic control can only be achieved by direct sensing of blood glucose concentration that is immediately coupled to insulin secretion (and perhaps glucagon) into the blood stream (Fig. 1). This can now only be achieved by fully functional beta cells that are vascularized. Therefore, current immunoprotective micro- and macro-encapsulation strategies preclude optimal insulin dynamics, but may still have relevant beneficial effects for glycemic control in patients. Looking further ahead in the future, gene editing strategies creating donor cells that are tolerated by the immune system, and other immuno-evasive strategies that allow vascularization, would provide an optimal solution [59, 60].

Thus, in the interest of patients with diabetes mellitus, the two races to reach optimal glycemic control at a minimal risk and burden for patients should continue at full speed. While developments on the artificial pancreas are likely to lessen the burden of diabetes and improve quality of life in the short term, novel beta-cell replacement strategies should be developed with the ultimate aim of completely normalizing glycemic control in the future.
